# Supplementary material for: Activation of store-operated calcium entry and mitochondrial respiration by enterovirus 71 is essential for efficient virus replication
Source: mBio. 2025 Jul 8;16(8):e03717-24. doi: 10.1128/mbio.03717-24 (PMC12345184; doi:10.1128/mbio.03717-24)
Supplement: Supplemental figures — Fig. S1 to S8. [file mbio.03717-24-s0002.pdf]

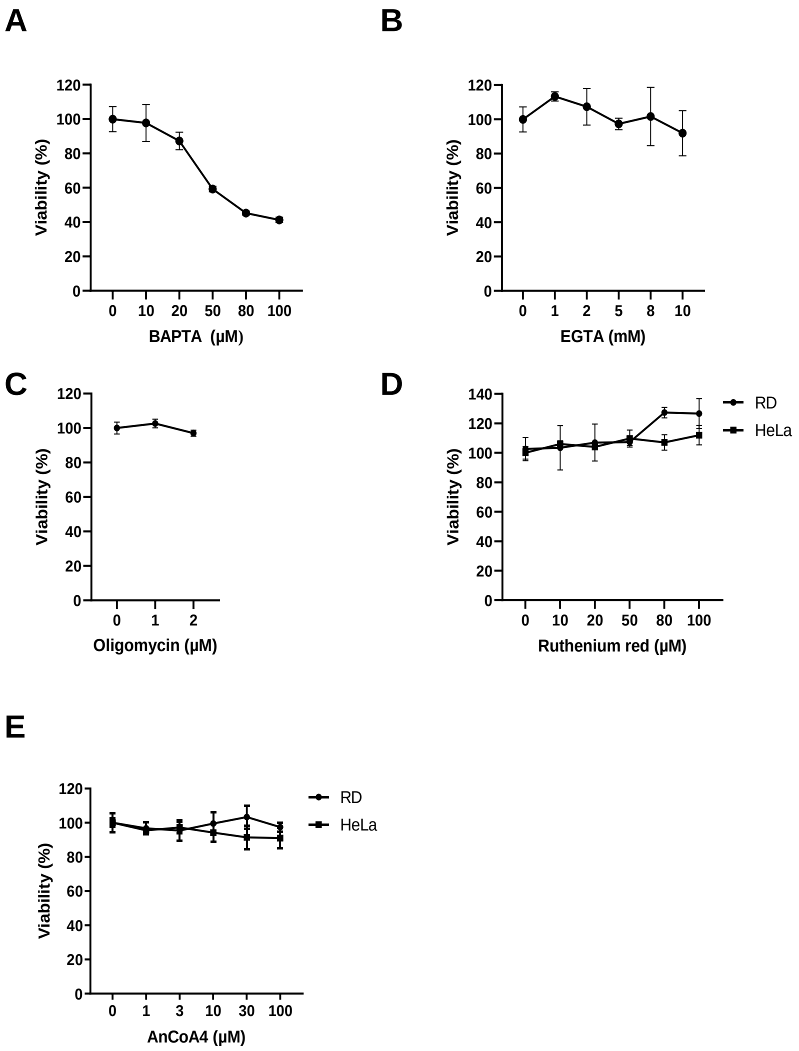

Fig S1 Cell viability upon the test compound treatments. RD cells were treated with (A) BAPTA, (B) EGTA, (C) oligomycin at concentrations indicated for 12 h. RD and HeLa cells were treated with ruthenium red (D) or AnCoA4 (E) at concentrations indicated for 24 h. Levels of cell viability were determined by MTS assay (See Text S1), and shown as relative levels to those of DMSO treated control cells. Triplicated experiments were conducted, and shown as means ± SDs.

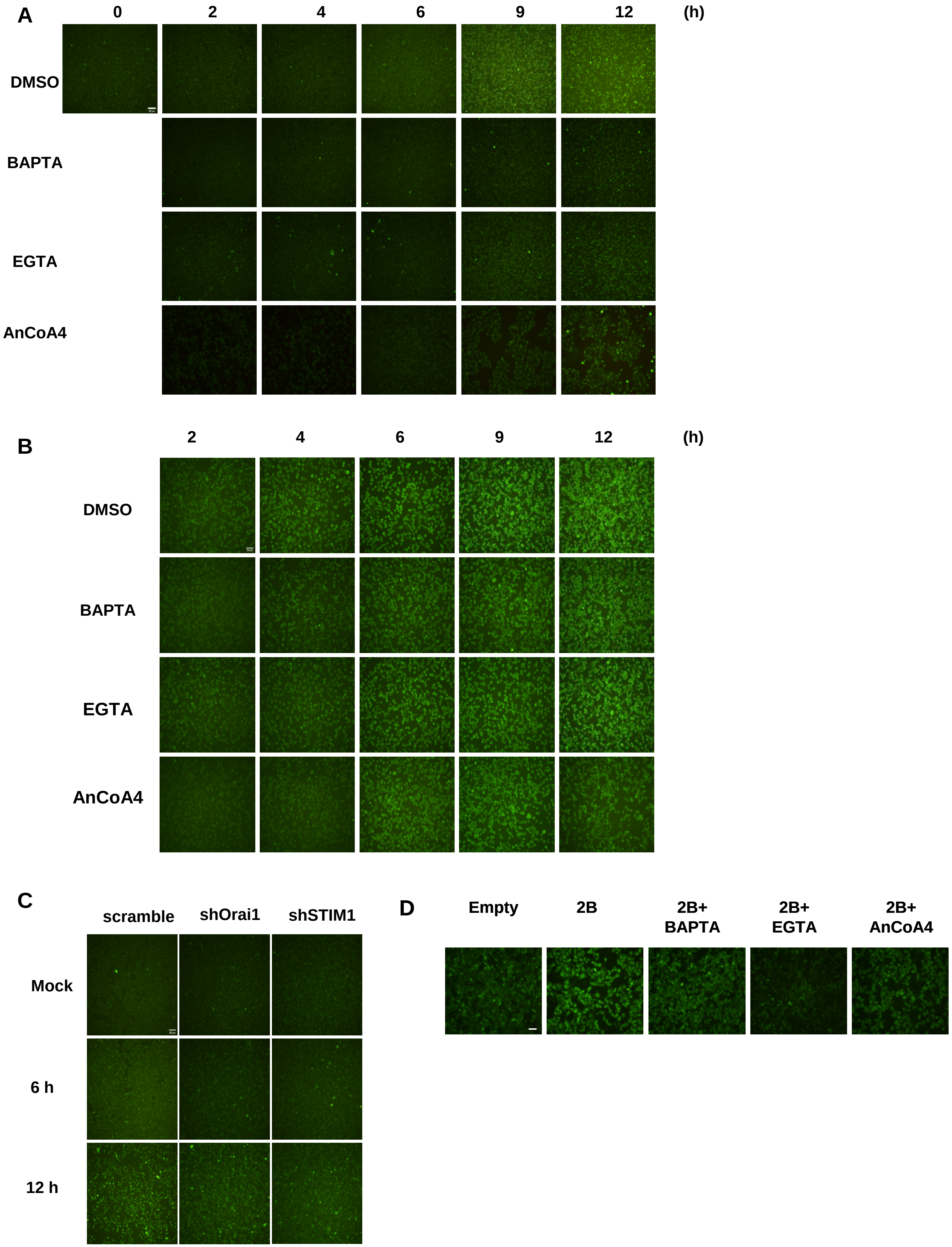
A
0
2
4
6
9
12
(h)
DMSO
BAPTA
EGTA
AnCoA4
B
2
4
6
9
12
(h)
DMSO
BAPTA
EGTA
AnCoA4
C
scramble
shOrai1
shSTIM1
Mock
6 h
12 h
D
Empty
2B
2B+ BAPTA
2B+ EGTA
2B+ AnCoA4

Fig S2 Image of cytosolic Ca^{2+} fluorescence upon the treatments indicated. (A, B) RD cell were infected with EV71 at an MOI of 0.5(A) or 5 (B) for the indicated times, and incubated with BAPTA, EGTA, AnCoA4 or DMSO control for the infection periods. (C) HeLa cells were transduced with lentivirus–shScramble, –shOrai1 or –shSTIM1 48 h prior to infection with EV71 at an MOI of 0.5 for indicated times. (D) HeLa cells were transfected with pFLAG-CMV-2 (empty) plasmid, pCMV-FLAG-2B (2B) plasmid, or left untransfected (Unt) for 40 h. For untransfected and empty controls, media were replaced with DMSO-containing media, and incubated for 8 h. For the pCMV-FLAG-2B -transfected cells, media were replaced with DMSO-, EGTA-, or BAPTA-containing medium, as indicated, and also incubated for 8 h. At the end of viral infections, cells were stained with Fluo-8 for 30 mins. Scale bars represented 50 μm.

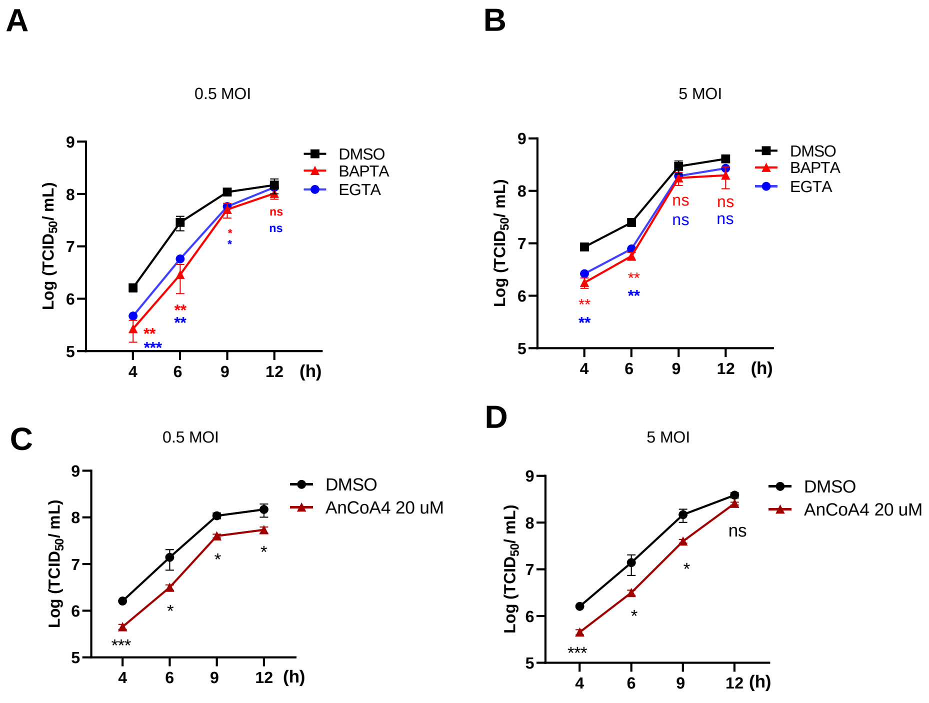

FIG S3 Effect of calcium chelators and SOCE blocker in HeLa cells during EV71 infection. HeLa cells were infected with EV71 stock at an MOI of 0.5 (A, B) or 5 (C, D) for the indicated times in the presence of BAPTA (10 µM), EGTA (1.8 mM) (A, B), AnCoA4 (20 µm)(C, D) or DMSO control. Cell lysates and supernatants were collected at the indicated times. Total virus titers were determined by $TCID_{50}$ assay. Data represent mean values from experiments conducted in triplicate, with error bars indicating standard deviations (SDs). ***p < 0.001, **p < 0.01, *p < 0.05 versus the DMSO control.

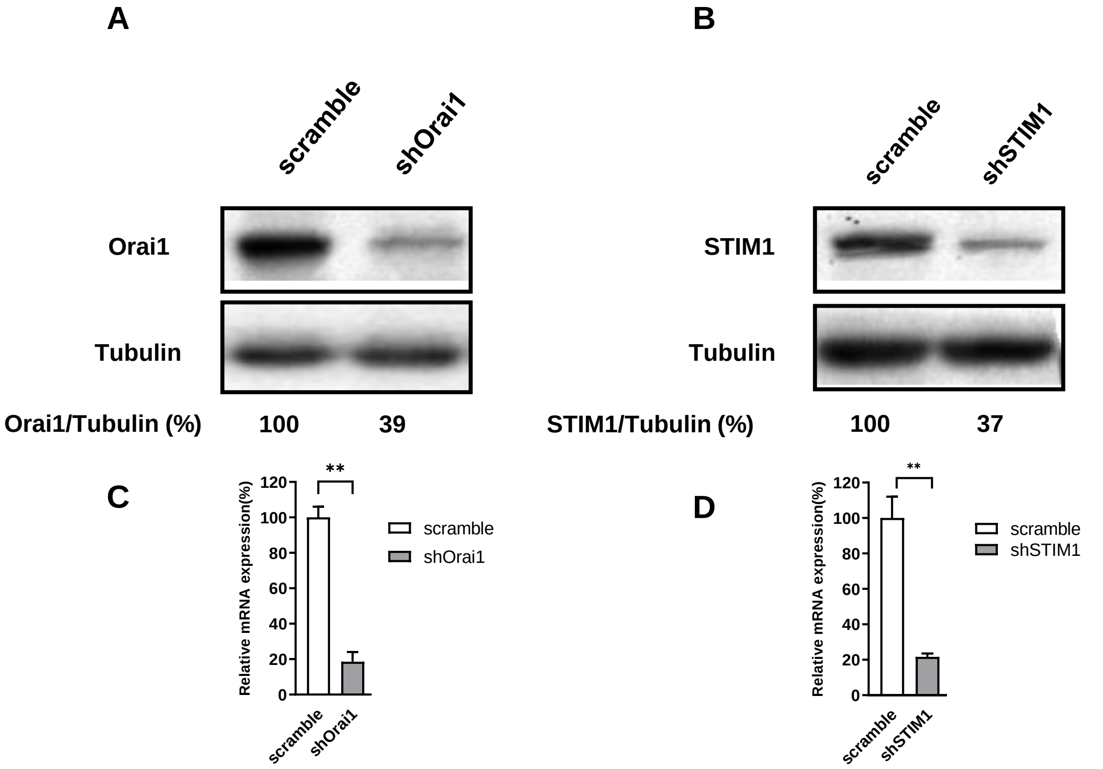

Fig S4 Validation of knockdown efficiency using immunoblot analysis and RT-qPCR. HeLa cells with knockdown of Orai1 (A and C) or STIM1 (B and D), along with scramble controls, were collected to prepare cell lysates (A and B) and total RNA (C and D) for assessing knockdown efficacy. Immunoblot analysis was performed using anti-Orai1 antibodies (A) and anti-STIM1 antibodies (B), with anti-tubulin antibodies serving as the internal control. The percentages shown below each lane represent the intensity of Orai1 or STIM1 relative to that of tubulin. The relative RNA levels of Orai1 (C) and STIM1 (D) were quantified through RT-qPCR, using GAPDH as the internal control. Statistical significance is indicated as follows: ***p < 0.001, **p < 0.01, *p < 0.05 compared to scramble controls.

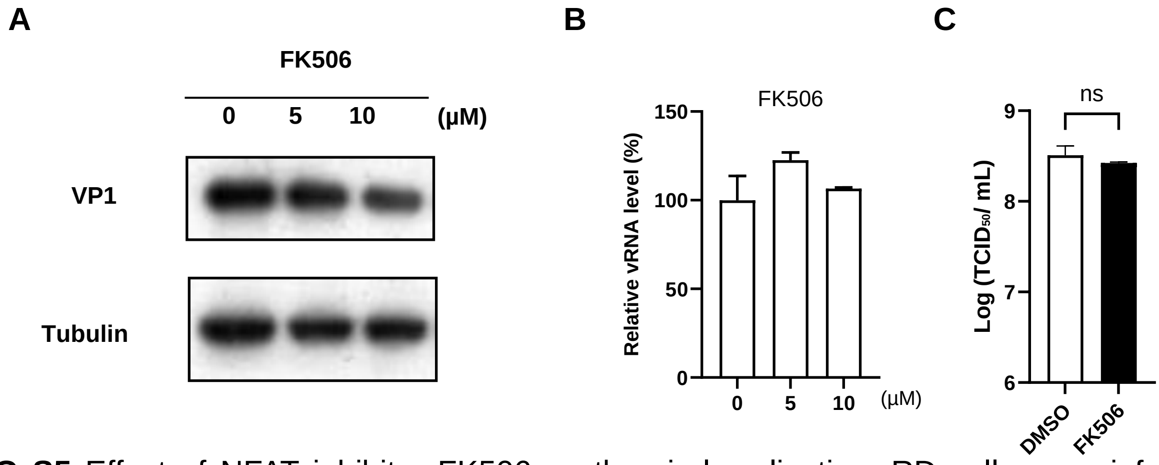

FIG S5 Effect of NFAT inhibitor FK506 on the viral replication. RD cells were infected with EV71 at an MOI of 0.5, and treated with FK506 at 5- and 10- μM, or DMSO control. Cell lysates were prepared for the Immunoblot analysis on viral VP1 protein and tubulin as the internal control. (B) Total RNA was prepared to measure the relative viral RNA levels by RT-qPCR analysis and (C) Total viral titers (see Text S1) were determined by $TCID_{50}$ assay.

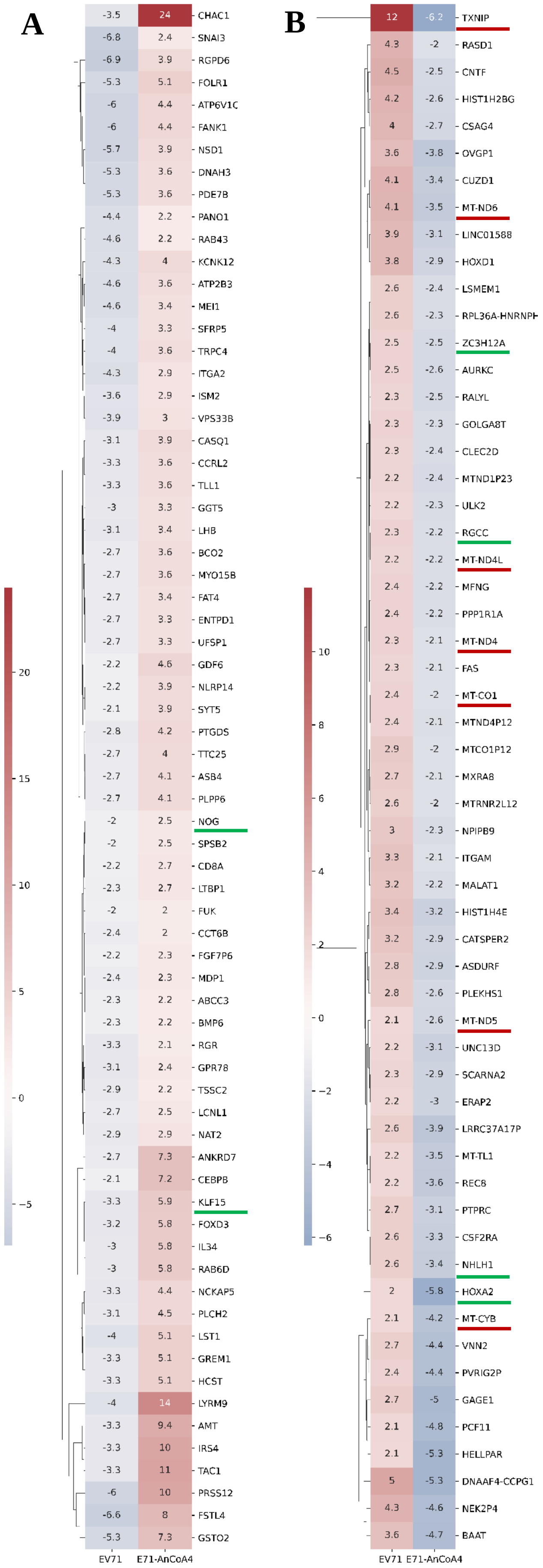

FIG S6 Heatmap and 2-dimension hierarchical clustering of differentially expressed genes (DEGs, fold change > 2) between the DMSO- and AnCoA4-treated groups, both in the context of EV71 infection at 0.5 MOI. The group that includes genes downregulated by EV71 infection yet upregulated upon AnCoA4 treatment (A), and those upregulated by EV71 infection yet downregulated upon AnCoA4 treatement (B) are shown, with the numbers for each gene indicating the change folds. For the DEGs listed in Table 1, those associated with mitochondrial ETC are underlined in red while those independent of mitochondrial ETC are underlined in green.

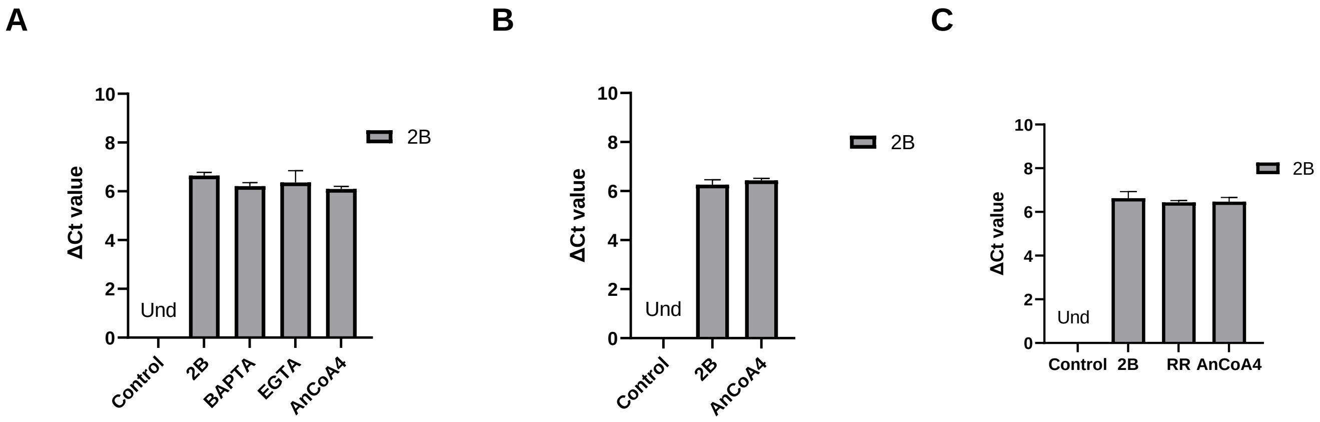

FIG S7 Validation of viral 2B expression in HeLa cells transfected with the 2B-expressing plasmid. HeLa cells were transfected with either the pFLAG-CMV-2 (empty control) plasmid or the pCMV-FLAG-2B (2B) plasmid. The expression levels were quantified using RT-qPCR as detailed in Text S1. Panels A, B, and C correspond to the conditions shown in Fig. 6A, Fig. 6B, and Fig. 8B, respectively. For each measurement, the ΔCt value was calculated by subtracting the Ct value of GAPDH from that of the 2B gene. The Ct values for the empty control were undetermined (Und, Ct > 40). The data are presented as the mean ± standard deviation of triplicate measurements.

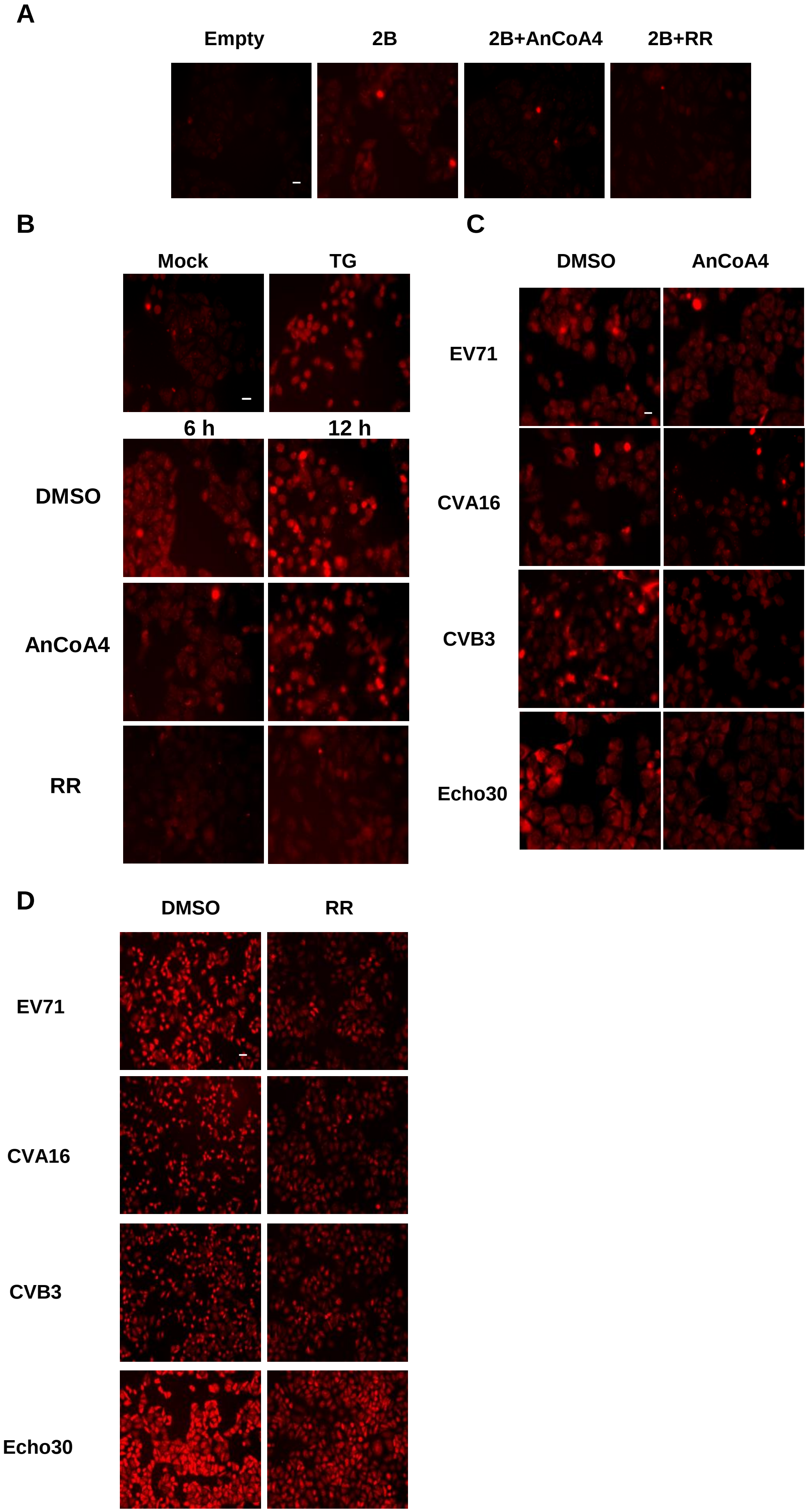
A
Empty
2B
2B+AnCoA4
2B+RR
B
Mock
TG
6 h
12 h
DMSO
AnCoA4
RR
C
DMSO
AnCoA4
EV71
CVA16
CVB3
Echo30
D
DMSO
RR
EV71
CVA16
CVB3
Echo30

FIG S8 Image of mitochondrial Ca^{2+} fluorescence after the treatment of RR and AnCoA4. (A) HeLa cells were transfected with an empty or 2B-expressing plasmid for 40 h. The media were replaced with DMSO, 10 μM RR, or 20 μM AnCoA4-containing media for 8 h and then stained with Rhod-2. (B) HeLa cells were treated with 10 μM RR, 20 μM AnCoA4, or DMSO control during EV71 infection at an MOI of 0.5 for 6 h and 12 h and were then stained with Rhod-2. TG served as a positive control for Rhod-2 stain. (C, D) HeLa cells were infected with EV71, CVA16, CVB3, or Echo 30, each at an MOI of 0.5 for 6 h, and were treated with 20 μM AnCoA4 (C), 20 μM RR (D), or left untreated (DMSO treatment) during the infection period. The cells were stained for Rhod-2. The scale bar represented 50 μm.
