# Supplementary material for: Activation of store-operated calcium entry and mitochondrial respiration by enterovirus 71 is essential for efficient virus replication
Source: mBio. 2025 Jul 8;16(8):e03717-24. doi: 10.1128/mbio.03717-24 (PMC12345184; doi:10.1128/mbio.03717-24)
Supplement: Supplemental text — Text S1. [file mbio.03717-24-s0003.pdf]

Supplemental Materials and Methods (Text S1)

Chemicals

1,2-bis(2-aminophenoxy) ethane-N,N,N',N'-tetraacetic acid (BAPTA-AM,100168, MedChemExpressm,Monmouth Junction, NJ), EGTA (324626,Merck,Whitehouse Station, NJ), thapsigargin (10522,Cayman), AnCoA4 (AOB6289, AOBIOUS, Gloucester, MA), Ruthenium red (557450, Merck) and oligomycin (13995, Cayman, Ann Arbor, MI) were dissolved in DMSO. All DMSO control samples contained 0.05 % DMSO. Fluo-8 NW (36307, AAT Bioquest,Sunnyvale, CA) and Rhod-2-AM (R1244, Invitrogen, Carlsbad, CA) stocks were dissolved in PBS.

Virus infection

RD or HeLa cells were plated in a 6-well overnight and were grown to approximately 80% confluence prior to EV71 infection. The virus was diluted in 1 mL of minimum essential medium (MEM) supplemented with 2 % fetal bovine serum (FBS) (MEM-2) and incubated with the cells at the indicated multiplicity of infection for 1 h at 37°C. Then, the unbound virus was removed by washing three times with PBS and the cells were incubated with 2% FBS MEM containing the aforementioned chemicals for the time periods indicated.

Virus titration

The supernatant containing extracellular viruses was collected from the EV71 infected RD cell culture and centrifugated at 2,000 × g for 5 min to remove cell debris. Intracellular viruses were prepared from cell lysates collected after freeze-and-thaw cycles three times and centrifugation at 13,000 × g for 10 min. Total viruses were obtained by combining intracellular and extracellular viruses in a 1:1 ratio. The infectious viral titer was measured using fifty-percent tissue culture infective dose ($TCID_{50}$) according to the Reed-Muench method.

Time-of-addition assay

AnCoA4 at 20 μM was added to the RD cells at different time points relative to virus inoculation at 0.5 MOI as described below. (I) Pretreatment of the cells with AnCoA4 and maintained in the cultures throughout the infection period. Cells were treated with AnCoA4 for 1 h (-1-0 h). After washing with PBS to remove AnCoA4, the cells were inoculated with virus (0-1 h, viral adsorption) in the presence of AnCoA4 (0-1 h) for another 1h. After the residual virus was removed, the cells were cultured in MEM-2 containing AnCoA4 for 6 h. (II) Pretreatment of the cells with AnCoA4 and maintained in the cultures for the viral entry stage. Cells were treated with AnCoA4 for 1 h (-1-0 h). After washing with PBS to remove AnCoA4, the cells were inoculated with virus in the presence of AnCoA4 (0-1 h) for another 1h. (III and IV) Post-inoculation treatment of virus-infected cells with AnCoA4. Cells were inoculated with virus in the absence of AnCoA4 for 1h. After 1h of viral adsorption, the cells were washed with PBS to remove the virus, and AnCoA4 was added during 1-3 h (III) and 3-6 h (IV).

Construction of plasmid

To construct 2B-bearing plasmids, total RNA from EV71 virus stock was prepared. Reverse transcription-PCR was conducted to amplify 2B - (nt 3780-4076 of EV71 BrCr strain) coding region by the primer pairs with engineered *Bam*HI/*EcoRI* sites (Supplementary Table 1). These PCR products were subsequently digested and directionally cloned into the *BamHI*/*EcoRI* sites of plasmid pFLAG-CMV-2 (Sigma-Aldrich), generating the 2B-bearing plasmids designated pCMV-FLAG-2B.

Transfection of HeLa cells

HeLa cells (2×10^6 cells) were seeded in a 6-cm dish, and transfected with the pFLAG-CMV-2 plasmid or the pCMV-FLAG-2B plasmid using TransIT-LT1 transfection reagent (MIR2300, Mirus). Briefly, 5 µg plasmid DNA was complexed with 15 µl transfection reagent in 400 µl Opti-MEM® (31985062, Gibco) and incubated at room temperature for 30 min. The transfection mixture was added to cell monolayers and incubated for 24 h. Subsequently, transfected cells were trypsinized and divided into wells in a 24-well plate to maintain consistent transfection efficiency. Cells were subsequently incubated with either DMSO or test compounds for 8 h. The expression levels of 2B in the experimental groups were quantified through RT-qPCR.

Confocal microscopy of Orai1 and STIM colocalization

The pEGFP-STIM1 plasmid was used to transfect HeLa cells for 48 h, and the cells were selected using 1 µM puromycin for 48 h. Subsequently, the cells were sorted using a flow cytometer (FACS ARI, BD Biosciences) with a 488-nm laser and were designated as HeLa-EGFP-STIM1. Next, the pmOrange-Orai1 plasmid was used to transfect the HeLa-EGFP-STIM1 cells for 48 h to obtain cells co-expressing STIM and Orai1; these were designated HeLa-STIM1-Orai1 cells. The cells were fixed with 4% (PFA) and permeabilized using 0.2% Triton X-100. The primary antibody (Ab) that was used was mouse anti-EV71 (1:1000; MAB979, Merck), and the secondary Ab was anti-mouse Ab conjugated with cyanine 5 (Cy5, 1:100, ab52061, Abcam). The cell nuclei were stained with DAPI (ab228549, Abcam) at 10 mM. The cells were observed using a confocal microscope (Zeiss LSM 880) at 350, 488, 543, and 650 nm for DPAI, EGFP, mOrange, and Cy5, respectively. A comprehensive tool for quantitative colocalization analysis is an ImageJ plugin termed JACoP (for just another colocalization plugin) (PMID: 17210054). Images were performed colocalization analysis with JACoP to calculate Mander's Coefficient (PMID: 33930978). Mander's coefficient showing proportion of mOrange-Orai1 colocalized with EGFP-STIM1.

RNAi vector generation and target cell transduction

The lentivirus-based, short hairpin RNA (shRNA) constructs pLKO-shOrai1 (TRCN0000163585), pLKO-shSTIM1 (TRCN0000146686), targeting Orai1 and STIM1, respectively, and the negative control pLKO-shScramble (ASN0000000001) were purchased

from the National RNAi Core Facility, Academia Sinica, Taiwan. For lentivirus preparation, 293T cells were cotransfected with a pLKO-shRNA construct and two helper plasmids, VSV-G pMD.G and pCMVDR8.91, following instructions of the National RNAi Core. HeLa target cells were transduced at a multiplicity of infection (MOI) at 5 in the presence of 8 μg/mL phosphatidylserine and selected with 1 mg/ml puromycin for 48 h to generate stable knockdown or control cells.

Immunoblot

Immunoblots were conducted following the procedure in a prior study (Tseng KC, 2022). Primary antibodies (Abs) used were mouse anti-EV71 VP1 (1:1000, PAB0096, Abnova), rabbit anti-α tubulin (1:5000, GTX112141, GeneTex), mouse anti-Orai1 (1:1000, 13130-1-AP, Proteintech), and rabbit anti-STIM1 (1:2000,11565-1-AP, Proteintech). Horseradish peroxidase (HRP)-conjugated goat anti-mouse monoclonal Ab (1:2000, PAB0096, Abnova) or goat anti-rabbit HRP-conjugated Ab (1:5000, GTX213110-01, Gentex) was used as a secondary Ab. Protein levels were detected using an enhanced chemiluminescence (ECL) Western blot kit (Perkin Elmer).

Immunofluorescence assay

The immunofluorescence assay was conducted followed the procedure described previously (Tseng et al., 2022). RD cells were fixed with 4% PFA and penetrated using 0.2% Triton X-100. Cells were washed by phosphate buffered saline (PBS) and blocked by 3% Bovine Serum Albumin (BSA) for 1 h. Abs were used to probe several EV serotypes: mouse anti-EV71 (1:1000; MAB979, Merck) for EV71 and CVA16; mouse anti anti-coxsackie virus B blend (1:1000; MAB9410, Chemicon) for CVB3; mouse anti-echovirus blend Ab (1:1000; MAB9670, Merck) for Echo 30. A fluorescein (FITC)-conjuated goat anti-mouse IgG (1:100; cat. no. 115-095-062, Jackson ImmunoResearch) served as a secondary Ab. The cell nuclei were counterstained with DAPI. Cells were observed by a fluorescent microscope (Leica DM6000B) equipped with FITC and UV filters. Viral antigen-positive cells and the nuclei were quantified using Metamorph software, and the antigen-positive cell numbers were normalized by the corresponding nucleus numbers. Data are expressed as viral antigen (%) obtained in compound-treated cells relative to the infected, compound-free cells.

RNA extraction and RT-qPCR

Total RNAs from RD or HeLa cells were extracted by the TRIzol reagent (RN0200, GOAL Bio, Taiwan). RNAs from the mouse organoids were extracted by RNeasy Kits (Qiagen). Samples were reverse transcribed into cDNA using High-Capacity cDNA Reverse Transcription Kit (4368814, Applied Biosystems). quantitative PCR (qPCR) was performed using Fast SYBR Green Master Mix (4385616, Applied Biosystems) and Ct values were analyzed by StepOnePlus Real-Time. PCR System (Applied Biosystems). The gene expression levels were normalized using human GAPDH for RNAs from human cells (RD and HeLa cells), and with mouse GAPDH for those from mouse intestinal

organoids. The sequences of primers were listed in the Table S1.

Measurement of mitochondrial complex I activity

To analyze the activity of mitochondrial complex I (NADH dehydrogenase), we isolated mitochondria from HeLa cells by using the Mitochondria Isolation Kit (ab110170, Abcam) followed by the Complex I Enzyme Activity Assay Kit (ab109721, Abcam) in accordance with the manufacturer's instructions. Complex I activity was determined by measuring the oxidation of NADH to NAD^+ and the simultaneous reduction of a dye, with increased absorbance occurring at 450nm, which was measured using the microplate reader (Infinite 200 PRO, TECAN).

ATP measurement

The ATP levels in the cells were determined using a luciferin-luciferase-based assay in accordance with the manufacturer's instructions. Briefly, the cells were seeded in 96-well microplates and were lysed and processed using an ATPlite Luminescence Assay system (016943, PerkinElmer, Wellesley, MA). Luminescent signals from each well were measured using the microplate reader.

Cell viability assay

Cell viability was determined by using the MTS Assay Kit (ab197010, Abcam) according to the manufacturer's instructions, and the absorbance measured by TECAN Infinite 200 PRO plated reader at 490 nm.

Caspase activity assay

The Caspase-3, -8, -9 Activity Assay Kit (ab39383, ab39534, ab65607, Abcam) was used, and the assays conducted according to the manufacturer's protocol. Briefly, cell lysates (25 μg) were added to the fluorogenic substrates, and incubated at 37 °C for 2 h. Fluorescence intensities were measured by spectrophotometry using TECAN Infinite 200 PRO plate reader.

Isolation of intestinal stem cell from mice

Intestinal tissues from C57BL/6JNarl mice aged 4 to 6 weeks were cut into 0.6cm fragments and then soaked in ice-cold PBS containing gentamicin (0.5 mg/mL). This mixture was gently shaken at 4°C for 10 min to remove feces and contaminants. The fragments were then transferred to 5 mL of PBS with added $CaCl_2$ (100 mg/L) and $MgCl_26H_2O$ (100 mg/L) and shaken at 75 rpm for 20 min at 4°C. After the PBS supernatant was removed, we introduced 5 mL of PBS with added EDTA (1 mM) and EGTA (1 mM) to the tube. The mixture was shaken at 75 rpm for 10 minutes at 4°C to eliminate the villus fractions. After the PBS supernatant was remove, the fragments were immediately vortexed for mixing. The cells suspended in PBS were collected as fraction 1. To convert the cluster fractions into individual crypt cell populations, the PBS incubation and vortex steps were repeated seven times until fraction 8 was obtained. Fractions 6 to 8 were filtered through a cell strainer and then centrifuged at 900 rpm for 10 minutes at 4 °C The resulting fractions were resuspended using crypt medium.

Imaging of crypt organoids

Organoids were fixed with 4% PFA for 2 h, followed by permeabilization with 0.25 % Triton X-100 treatment. Organoids were incubated Anti-EV71 Ab (1: 1000; Sigma-Aldrich, MAB 979) for 48 h at 4°C. Organoids were then washed with dilution buffer. Anti-mouse FITC (1: 100; 115-095-062, Jackson ImmunoResearch) was added and incubated for 2 h. Then organoids were soaked in Focus Clear (FC01, CelExplorer Lab) for overnight at 4°C. Cell nuclei were stained by DAPI mounting solution (ab104139, Abcam). The images of crypt organoids were taken by confocal microscopy (Zeiss LSM 880). The quantification of florescence intensity was performed by software LSM 880 Zen blue.

Ethics Statement

This study was approved by the Ethic Committee of Far Eastern Memorial Hospital.

Raw data from RNA-seq is provided in the supplemental material
