# Supplementary material for: Activation of store-operated calcium entry and mitochondrial respiration by enterovirus 71 is essential for efficient virus replication
Source: mBio. 2025 Jul 8;16(8):e03717-24. doi: 10.1128/mbio.03717-24 (PMC12345184; doi:10.1128/mbio.03717-24)
Supplement: Legend — Data Set S1 legend. [file mbio.03717-24-s0004.pdf]

Data Set S1

Differentially expressed genes in the RD cells under the following conditions: mock-infected (RD-Mock), EV71-infected with DMSO treatment (RD-EV71), and EV71-infected with AnCoA4 treatment (EV71-AnCoA4). Columns are as follows: Gene Name, Gene length, Gene ID, Gene description, Fold change, FPKM reads, and Total exon fragments.
