# Supplementary material for: Activation of store-operated calcium entry and mitochondrial respiration by enterovirus 71 is essential for efficient virus replication
Source: mBio. 2025 Jul 8;16(8):e03717-24. doi: 10.1128/mbio.03717-24 (PMC12345184; doi:10.1128/mbio.03717-24)
Supplement: Table S1 — Primers. [file mbio.03717-24-s0005.pdf]

Table S1 Sequences of primers for cloning and RT-qPCR

Primer name	Primer Sequences (5'-3')
EV71-2B-forward-cloning	GCGAATTCGGGGGTATCTGATTACATCAA
EV71-2B-reverse-cloning	CAGGATCCTTACTGTTTTTGTGCCATGGGAA
EV71-2B-forward-qPCR	CGGGGGTATCTGATTACATCAA
EV71-2B-reverse-qPCR	CTGTTTTTGTGCCATGGGAA
EV71 VP1 forward	AGTATGATTGAGACTCGGTG
EV71 VP1 reverse	GCGACAAAAGTGAACTCTGC
STIM1 forward	CAGTGAAACACAGCACCTTC
STIM1 reverse	AAGAGCACTGTATCCAGAGCC
Orai1 forward	ACCTCGGCTCTGCTCTCC
Orai1 reverse	GATC TGAGCGCAAACAGG
Human GAPDH forward	CACCACCATGGAGAAGGCTGG
Human GAPDH reverse	CCAAAGTTGTCATGGATGACC
MT-ND4 forward	TCTTCTTCGAAACCACACTT
MT-ND4 reverse	AAGTACTATTGACCCAGCGA
MT-ND6 forward	CAAACAATGTTCAACCAGTAACCACTAC
MT-ND6 reverse	ATATACTACAGCGATGGCTATTGAGGA
MT-CYB forward	ATCACTCGAGACGTA AATTATGGCT
MT-CYB reverse	TGAACTAGGTCTGTC CCAATGTATG
EV71 MP4 forward	GAGAGTTCTATAGGGGACAGT
EV71 MP4 reverse	AGCTGTGCTATGTGAATTAGGAA
Mouse mt-ND6 forward	CTTGA TGGTT TGGGA GATTG G
Mouse mt-ND6 reverse	ACCCG CAAAC AAAGA TCACC
Mouse mt-CYB forward	ACACG CAAAC GGAGC CTCAA
Mouse mt-CYB reverse	TGCTG TGGCT ATGAC TGCGA ACA
Mouse mt-COI forward	CCAGT GCTAG CCGCA GGCAT
Mouse mt-COI reverse	TCTGG GTGCC CAAAG AATCA GAACA
Mouse GAPDH forward	TGCACCACCAACTGCTTA
Mouse GAPDH reverse	GGATGCAGGGATGATGTTC
